# Amino Acid Fingerprinting of Authentic Nonfat Dry Milk and Skim Milk Powder and Effects of Spiking with Selected Potential Adulterants

**DOI:** 10.3390/foods11182868

**Published:** 2022-09-16

**Authors:** Sneh D. Bhandari, Tiffany Gallegos-Peretz, Thomas Wheat, Gregory Jaudzems, Natalia Kouznetsova, Katya Petrova, Dimple Shah, Daniel Hengst, Erika Vacha, Weiying Lu, Jeffrey C. Moore, Pierre Metra, Zhuohong Xie

**Affiliations:** 1 Merieux NutriSciences, 3600 Eagle Nest Drive, Crete, IL 60417, USA; 2 FutureCeuticals, 2692 N. State Rt. 1-17, Momence, IL 60954, USA; 3 Waters Corporation, 34 Maple Street, Milford, MA 01757, USA; 4 Nestlé Quality Assurance Center, 6625 Eiterman Rd., Dublin, OH 43017, USA; 5 United States Pharmacopeia (USP), 12601 Twinbrook Parkway, Rockville, MD 20852, USA; 6 Eurofins Food Integrity and Innovation, Madison, WI 53704, USA; 7 Institute of Food and Nutraceutical Science, Department of Food Science and Technology, School of Agriculture and Biology, Shanghai Jiao Tong University, Shanghai 200240, China; 8 Merieux NutriSciences Corporation, 113 Route de Paris, 69160 Tassin la Demi-Lune, France

**Keywords:** milk powder, authentication, adulteration, amino acids, microwave-assisted hydrolysis, chromatography

## Abstract

A collaborative study was undertaken in which five international laboratories participated to determine amino acid fingerprints in 39 authentic nonfat dry milk (NFDM)/skim milk powder (SMP) samples. A rapid method of amino acid analysis involving microwave-assisted hydrolysis followed by ultra-high performance liquid chromatography-ultraviolet detection (UHPLC-UV) was used for quantitation of amino acids and to calculate their distribution. The performance of this rapid method of analysis was evaluated and was used to determine the amino acid fingerprint of authentic milk powders. The distribution of different amino acids and their predictable upper and lower tolerance limits in authentic NFDM/SMP samples were established as a reference. Amino acid fingerprints of NFDM/SMP were compared with selected proteins and nitrogen rich compounds (proteins from pea, soy, rice, wheat, whey, and fish gelatin) which can be potential economically motivated adulterants (EMA). The amino acid fingerprints of NFDM/SMP were found to be affected by spiking with pea, soy, rice, whey, fish gelatin and arginine among the investigated adulterants but not by wheat protein and melamine. The study results establish an amino acid fingerprint of authentic NFDM/SMP and demonstrate the utility of this method as a tool in verifying the authenticity of milk powders and detecting their adulteration.

## 1. Introduction

Milk is nature’s most complete food [1], playing an important role in the diet of over 6 billion people [2,3,4]. Milk and other dairy products are important sources of nutrients for human health [5]. In recent decades, milk product consumption has rapidly increased in several developing countries, particularly in parts of East and Southeast Asia [6]. The world consumption of dairy products is expected to increase over the coming decade due to strong demand in India, Pakistan, and Africa, driven by increases in income and population growth. The consumption preferences of developed countries tend towards processed products, while in developing countries fresh dairy products comprise over 75% of average per capita dairy consumption in milk solids [7].

The production of dairy products has been increasing but still is not able to meet the rising consumer demand at affordable prices worldwide. This gap in supply and demand may be one of the driving forces behind adulteration of milk by producers and others involved in its distribution [8]. The global dairy market was valued at 720 billion U.S. dollars in 2019 and is projected to grow to about 1032 billion U.S. dollars by 2024 [9]. Milk powder is one of the most widely traded food commodities, with over 2.5 million metric tons exported annually [10]. The milk powders are not only used for recombination or reconstitution for nutritional purposes, but also for their intrinsic functional properties [11]. The production and sales of dairy foods in mass volumes and their premium prices based on the composition also add motivation for adulteration. The lack of suitable methods to detect adulteration further aggravates the problem and adds to the incentive for economically motivated adulteration (EMA) of milk and other dairy products [12]. The addition of melamine to infant milk powder reported in 2008 in China, is one example of EMA. Melamine ended up in infant formulas from the adulterated milk as a substitute to increase its nitrogen content [13]. The motivation for food fraud is often economic, but its impact is a real public health concern [8,14,15]; in the case of the melamine scandal, six children died and several thousands more were hospitalized [16].

The value of milk powder is linked to its protein content; standard methods for protein analysis rely on a simple nitrogen assay [17]. Currently, the testing standards that are widely used by food manufacturers are not able to differentiate dairy protein from non-protein nitrogen and other proteins, which unfortunately provides fraudsters with opportunities to boost the economic value of ingredients by adding inferior, nitrogen-rich compounds [18,19,20]. This may lead to the addition of cheap chemicals and industrial ingredients rich in nitrogen to artificially increase the apparent protein [21]. Moore et al. [17] identified limitations of current standard methods of protein analysis when applied to food protein authentication and detection of food protein adulteration and highlighted the potential utility of amino acid compositional analysis in these applications.

Amino acid fingerprints have been used in detection of food adulteration in a variety of food matrices. Cotte et al. (2004) [22] used amino acid fingerprint to discriminate different botanical origins of honey. It has also been used to determine authenticity of some fruit juices [23] and in differentiating various meats [24], and separating milk from non-milk proteins [25], and detection of adulteration in marine powders [26]. Recently, it has been used to identify the geographical origin of milk samples [27]. Amino acid fingerprints in these studies have been demonstrated to be useful in authentication of some food matrices but systematic studies in this regard are required for its application in authentication of milk powder samples.

The United States Pharmacopeial Convention (USP) has led many international collaborative research projects to develop a toolbox of screening methods and reference standards for authenticating milk powder samples and detecting their adulteration [28]. Test methods have been developed by volunteer experts in several diverse analytical areas, including wet chemistry (e.g., non-protein nitrogen detection) [29]; spectroscopy (e.g., NIR characterization) [30,31,32,33]; and chromatography (e.g., non-targeted analysis) [34]. Amino acid analysis helps to confirm that the nitrogen measurements correspond to true protein [17]. Amino acid fingerprints have been shown in earlier studies to be able to segregate milk proteins from certain non-milk proteins [28] and possibly can be hypothesized to differentiate between nonfat dry milk (NFDM)/skim milk powder (SMP) and milk powders spiked with low-cost proteins and other nitrogenous substances used for EMA. A method of determination of the amino acid fingerprints of NFDM/SMP and its use in evaluating their authenticity has been published as a General Tests and Assays entry in the Food Chemicals Codex (FCC) [35]. Here we report the data and rationale for development of a rapid method of amino acid analysis. Details of the method of microwave-accelerated acid hydrolysis of proteins followed by amino acid fingerprint determination using ultra-high performance liquid chromatography (UHPLC) with UV detection are provided. We also report the amino acid fingerprints of authentic NFDM and SMP samples and those spiked with selected potential adulterants.

## 2. Materials and Methods

### 2.1. Study Design

The study was designed to determine the amino acid fingerprints of authentic NFDM and SMP samples as well as those spiked with selected potential adulterants. NFDM and SMP share most specifications. For the discussion in the manuscript, we consider them the same product.

Five laboratories participated in this collaborative study, four in the U.S. and one in China. Laboratories participating in the study included the following, Waters Corporation laboratory, Milford, MA, USA; USP, Rockville, MD, USA; Nestle NQAC, Dublin, OH, USA; Covance Laboratories/Eurofins, Madison, WI, USA, and Shanghai Jiao Tong University, Shanghai, China.

Each participating lab in the study analyzed eight core authentic NFDM/SMP samples and one NIST reference material. Each lab also analyzed an additional set of four to five authentic NFDM and/or SMP samples. This enabled inclusion of the broadest possible range of authentic NFDM/SMP samples in the study.

To evaluate the performance of the amino acid fingerprint methodology in detection of adulteration of NFDM/SMP samples, one of the authentic NFDM/SMP samples, was spiked separately with each of the eight potential adulterants investigated. Amino acid composition of adulterant spiked samples as well as non-spiked authentic NFDM/SMP samples was determined.

### 2.2. Authentic NFDM/SMP Samples

Thirty-eight authentic NFDM/SMP samples representing six countries and eight production locations were analyzed in the study. These samples were provided to each laboratory by USP, Rockville, MD 20852, USA. NIST SRM 1549a Milk Powder (NIST, Gaithersburg, MD, USA) was analyzed along with the NFDM/SMP samples. The certificates of analysis (COA) of the commercial milk powder samples provided information about the milk type (SMP or NFDM), product origin details (raw milk geographic origin), processing conditions (low-, medium- and high-heat spray drying), and chemical composition (percent moisture, fat, and protein). Details about each of the authentic milk powder samples are shown in Table 1.

### 2.3. Adulterant-Spiked NDFM/SMP Samples

One of the authentic NFDM/SMP samples (S091) (the base authentic NFDM/SMP), was spiked separately with one of the eight potential adulterants including plant/animal proteins and other nitrogenous substances, i.e., melamine and arginine. Each of the five participating laboratories analyzed the selected NFDM/SMP unspiked sample as well samples spiked separately with each of the eight adulterants at 3 or 4 concentration levels. All adulterants were supplied by USP. Details of the adulterants and their spiking levels studied are presented in Table 2.

### 2.4. Preparation of Authentic NFDM/SMP Samples

#### Sample Solution

An aliquot of about 30 mg of the sample was suspended in an appropriate hydrolysis tube assembly with 2.5 mL of internal standard solution and 2.5 mL of HPLC grade water with the aid of a micro stir bar. Borosilicate glass pressure vessels (pressure rated up to 600 psi) of 10 mL volume (catalog number 908035, CEM Corp., Matthews, NC, USA) were used along with silicone caps (catalog number 909210, CEM Corp.). Teflon coated micro stir bars (catalogue number 162810, CEM Corp.) were used for mixing.

### 2.5. Preparation of Adulterant Sample Solutions for Spiking the NFDM/SMP Samples

#### 2.5.1. Adulterant Spike Solutions

Separate aqueous solutions of each adulterant were prepared so that the required amount of the adulterant could be spiked into the NFDM/SMP sample. 

#### 2.5.2. Adulterant Spike Sample Solutions

About 30 mg of S091 (base milk powder), the required amount of the adulterant spike solution and HPLC water (balance of 2.5 mL after subtracting the amount of the spike solution) and 2.5 mL of internal standard solution were added in the hydrolysis tube assembly and mixed with the aid of a micro stir bar.

### 2.6. Protocol for Hydrolysis and Analysis of Sample Solutions

#### 2.6.1. Sample Hydrolysis Using Microwave

The sample solutions were hydrolyzed using a microwave hydrolysis instrument with optional auto sampler (Discover SP or Discover SP-D (CEM Corp.), following the parameters listed in Table 3. A temperature set point was programed at 160 °C using the fastest ramp to the temperature setting. The instrument typically operates between 50 and 80 psi. The power was selected to heat the sample to the set temperature in 1:30 (minutes:seconds) ± 15 s.

#### 2.6.2. Evaluation of Efficiency of the Microwave Protein Hydrolysis Method

The efficiency of the microwave method of hydrolyzing proteins was evaluated by comparing digestion of a casein sample using this method to hydrolysis of the same sample using a reference method for protein hydrolysis, AOAC method 982.30 [36]. The amino acid analysis in these studies was performed by the reference USDA/FSIS method MSS2 (1993) [37]. The method employs ion exchange chromatography with post-column derivatization with ortho-phthalaldehyde (OPA), followed by fluorescence detection.

### 2.7. Amino Acids Analyzed

Total amino acids in samples—including proteinogenic as well as those present as free—were analyzed in this study. The amino acids analyzed included those listed in standard solution (Section 2.8.2). Aspartic acid analyzed was sum of aspartic acid and asparagine as aspartic acid) (Asp). Similarly glutamic acid analyzed was sum of glutamic acid and glutamine as glutamic acid) (Glu). Hydroxyproline (Hyp) was not detected in any of the NFDM/SMP and NIST samples or the milk powder samples spiked with any of the adulterants except those spiked with fish gelatin at higher (0.15% and 0.3%) spiking levels. Hyp was also detected in neat fish gelatin samples. Results for Hyp are not reported in this study to focus on the amino acid fingerprints of milk powder samples themselves. The contents of sulfur-containing amino acids (cysteine/cystine and methionine) were not reported in most part of this study because they could be partially destroyed by acid hydrolysis and require another method of hydrolysis. However, methionine (Met) was analyzed in the studies of validation of efficiency of the microwave method of protein hydrolysis. In the later studies neither Met nor Hyp were analyzed.

### 2.8. Amino Acid Analysis

#### 2.8.1. Reagents

All chemicals and reagents used in the study were of analytical or HPLC grade with the highest purity. Ortho-phthalaldehyde (OPA) was obtained from Millipore Sigma, St Louis, MO, USA. Amino acid derivatization reagent, 6-aminoquinolyl-N-hydroxysuccinimidyl carbamate (AQC), part of the AccQ•Tag^TM^ Ultra Derivatization Kit (P/N 186003836), was obtained from Waters Corp., Milford, MA, USA.

#### 2.8.2. Standard Solutions

##### Amino Acid Standard Stock Solution

This was a quantitative mixture containing 2.5 μmol/mL of each of the following amino acids in 0.1 N HCl: L-alanine (Ala), L-arginine (Arg), L-aspartic acid (Asp), L-glutamic acid (Glu), L-glycine (Gly), L-histidine (His), L-isoleucine (Iso), L-leucine (Leu), L-lysine-HCl (Lys), L-phenylalanine (Phe), L-proline (Pro), L-serine (Ser), L-threonine (Thr), L-tyrosine (Tyr), L-valine (Val) (Amino Acid Standard H, Part# 20088, Thermo Fisher Scientific Inc., Rockford, IL, USA or WAT088122 from Waters Corp.). A separate stock standard solution of L-methionine (Met) (product# 64319, Millipore Sigma) was prepared whenever this amino acid was analyzed. Similarly, a separate stock standard solution of trans-4-hydroxyproline (Hyp) (product# H54409, Millipore Sigma) was prepared whenever this amino acid was analyzed.

##### Internal Standard Solution

Norvaline (2.50 µmol/mL) solution was prepared in 12M HCl (Part # N7627, Millipore Sigma). 

##### Working/Calibration Standard Solutions

The standard stock solution and internal standard solution, 100 µL of each, was mixed with 800 µL of 0.1N HCl to prepare 1000 μL of the solution (final concentration of each amino acid and internal standard = 250 nmol/mL). A separate calibration solution of Met (250 nmol/mL) containing internal standard (250 nmol/mL) was prepared by mixing 100 µL of Met stock standard and 100 µL of internal standard solution with 800 µL of 0.1N HCl to prepare 1000 μL of the solution whenever this amino acid was analyzed. Similarly, a separate calibration solution of Hyp (250 nmol/mL) containing internal standard (250 nmol/mL) was prepared by mixing 100 µL of Hyp stock standard and 100 µL of internal standard solution with 800 µL of 0.1N HCl to prepare 1000 μL of the solution whenever this amino acid was analyzed.

#### 2.8.3. Amino Acid Analysis Method

Amino acids in the hydrolysates were analyzed using AOAC method 2018.06 [38], in which amino acids are derivatized using 6-aminoquinolyl-*N*-hydroxysuccinimidyl carbamate (AQC) reagent, separated on a C18 UHPLC column, and detected with an ultraviolet (UV) detector. The details of the method are described in the following section.

##### Processing of Hydrolysate Solutions

An aliquot of 1.0 mL of the hydrolysate stock solution was combined with 1.0 mL of 6 N NaOH and 3.0 mL of 0.2 M HCl and filtered through a 0.45-µm PVDF membrane filter (Millipore Sigma) into an HPLC vial.

##### Derivatization

The sample hydrolysate solutions and the working standard solutions were derivatized with the AccQ•Tag ^TM^ Ultra Derivatization Kit (P/N 186003836, Waters Corp.) following a method consistent with the instructions provided by the manufacturer.

##### Chromatographic Analysis

Derivatized standard and sample solutions were transferred to HPLC vials and the amino acids were analyzed. The method used a commercially available, proprietary kit combined with UHPLC analysis by either Chromatographic system A or B as described in the following section.

##### Chromatographic System A

ACQUITY H-Class UPLC system (Waters Corp).

Mode: UHPLC

Detector: UV (260 nm)

Column: 100 mm × 2.1 mm column with octadecyl silane 

Stationary phase 1.7 µm particle size (AccQ-Tag^TM^) Ultra column (P/N 186003837, Waters Corp.).

Flow rate: 0.7 mL/min

Pre-injector volume: 100 µL

Injection volume: 1.0 µL

Wash-solvent pre-inject: 0 s

Wash-solvent post-inject: 6 s

Sample temperature: Ambient

Column temperature: 43 °C

Mobile phase Solution A: AccQ●Tag^TM^ Ultra Eluent A Concentrate (P/N 186003838. Waters Corp.)

Mobile Phase Solution B: Water and AccQ●Tag^TM^ Ultra Eluent B (P/N 186003839. Waters Corp.), (90:10, *v*/*v*)

Mobile Phase Solution C: Water

Mobile Phase Solution D: AccQ●Tag^TM^ Ultra Eluent B (P/N 186003839. Waters Corp.)

Mobile Phase: Gradients utilized of mobile phase solutions listed in Table 4.

##### Chromatographic System B

ACQUITY Binary UPLC system (Waters Corp.)

Mode: UHPLC

Detector: UV (260 nm)

Column: 100 mm × 2.1 mm column with octadecyl silane stationary phase, 1.7 µm particle AccQ-Tag^TM^ Ultra Column (P/N 186003837, Waters Corp.)

Flow rate: 0.7 mL/min

Injection volume: 1 µL

Sample temperature: Ambient

Column temperature: 55 °C

Weak needle wash: Acetonitrile and water (5:95, *v*/*v*)

Strong needle wash: Acetonitrile and water (50:50, *v*/*v*)

Mobile Phase Solution A: Water and AccQ●Tag^TM^ Ultra Eluent A Concentrate (95:5, *v/v*) AccQ●Tag^TM^ Ultra Eluent A Concentrate = (P/N 186003838. Waters Corp., Milford, Massachusetts, or equivalent

Mobile Phase Solution B: AccQ●Tag^TM^ Ultra Eluent B (P/N 186003839. Waters Corp.)

Mobile Phase: See below gradient table (Table 5).

### 2.9. Data Analysis

The percentage of each amino acid was calculated on an as-is basis using the following equation:

Result (g amino acid/100 g sample) *PU* = (*rU*/*rS*) × (*CS*/*CU*) × 10^−6^ × *F* × 100

*rU* = internal standard ratio (analyte peak area/internal standard peak area) obtained from the hydrolyzed/derivatized sample solution

*rS* = internal standard ratio (analyte peak area/internal standard peak area) obtained from the derivatized Working standard solution

*CS* = concentration of amino acids in the Working standard solution, corrected for purity based on the reference material label claim (pmol/μL)

*CU* = concentration of sample in hydrolysate solution (mg/mL)

10^−6^ = combined factor for pmol to mol conversion and the mg to g conversion in case of each amino acid

*F* = molecular weight of each amino acid.

The amount of amino acid in the working standard is provided as nmol/mL, which is first converted to moles and then to grams; these adjustments have been made in the above calculations.

The distribution of each amino acid in the sample was calculated as the percent of the sum of all amino acids analyzed in the sample (normalized format) by the following equation:

Distribution of each of amino acids in sample *PS*, as %

*PS* = (*PU*/∑*P*) × 100 

*PU* = percentage of amino acids on an as-is basis (calculated as stated above)

∑*P* = sum of percentage of amount of 15 amino acids on an as-is basis (*PU*)

Prediction limits for authentic NFDM/SMP: The estimations of lower and upper tolerance limits are based on approximate 95% confidence prediction bounds. These approximate limits were obtained using a *t* distribution approximation which can be interpreted as an approximation to the marginal posterior distribution of future results. The limits were calculated as mean ± *k* × SD, where mean is the grand mean of all results, SD is the root-sum of all variance components, and *k* is obtained as

$$k=t_{0.95,df}\cdot \sqrt{1+\frac{1}{df+1}}$$

where *t*_0.95,*df*_ is the 95th percentile of the Student’s t-distribution having *df* degrees of freedom. The fractional value for *df* was obtained using the Satterthwaite approximation.

Significant differences in means were detected using one-way ANOVA and post-hoc Dunnett’s test. Statistics were analyzed using Minitab software, version 19 (2020, Minitab LLC, State College, PA, USA). Statistical significance was defined at *p* ≤ 0.05.

The multivariate modelling, including the principal component analysis (PCA) and partial least squares-discriminant analysis (PLS-DA) were performed by a MATLAB R2019a (The MathWorks, Natick, MA, USA) in-house script.

## 3. Results and Discussion

### 3.1. Development of Microwave-Accelerated Protein Hydrolysis Method

The protein hydrolysis was performed using a microwave hydrolyzer because of its speed, ease of the use, as well as its rigorous control of hydrolysis conditions. The parameters (i.e., sample size, hydrolysis temperature and time) were optimized in preliminary studies. The efficiency of the microwave method in protein hydrolysis was evaluated by comparing the results of digestion of a casein sample by this method with that of the hydrolysis performed using the reference method for protein hydrolysis, the AOAC method 982.30 [36]. The results of the evaluation studies are presented in Table 6.

The results in Table 6 indicate that the values obtained for each of the amino acids analyzed in a casein sample using the microwave-accelerated hydrolysis technique were similar to the results obtained using the reference method. Amino acid values obtained by both methods were not significantly different except for aspartic acid (Asp, asparagine + aspartic acid as aspartic acid) which displayed a slightly higher value (7%) in case of the microwave method. These results suggest equivalency of the microwave-accelerated protein hydrolysis to the reference method for protein hydrolysis, which has been used for decades in the analysis of amino acids including proteinogenic amino acids. The amino acid values for casein obtained using the microwave method compared fairly well with values reported in the literature [39,40].

### 3.2. Amino Acid Fingerprint of Authentic NFDM/SMP Samples

Amino acid analysis in 39 authentic NDFM/SMP samples (Table 1) by five laboratories were analyzed in replicates. One of the samples was analyzed in 86 independent replicates, eight samples were analyzed in no less than 10 replicates each, six samples in 6 replicates each, one sample was analyzed in triplicate, 22 samples were analyzed in duplicate, and one sample was analyzed a single time (*n* = 274). The results were calculated in the normalized format (as percent of sum of contents of all) based on the concentration of amino acids in the sample. The normalization was conducted because there was a wide discrepancy among absolute amino acid concentrations in different laboratories and normalization improved the precision of the results. Results in Table 7 provide the average value of the distribution of each amino acid in NFDM/SMP samples, the corresponding values of standard deviation, % relative deviation (% RSD), range of distribution (minimum and maximum), total number of independent analyses, lower and upper tolerance prediction limits of distribution of amino acid based on standard deviation and coverage factor (*k*) values.

Amino acid distribution in NFDM/SMP samples showed a remarkably high level of glutamic acid (plus glutamine as glutamic acid), which accounted for about 22% of the sum of contents of all fifteen amino acids analyzed and present in the samples (Table 7). Pro, Leu, Lys and Asp (including asparagine as aspartic acid) each accounted for about 8–10% of all amino acids. Most of the other amino acids accounted for about 3–6% each of all amino acids. Gly contributed the least among all the amino acids at around 2%.

The obtained NFDM/SMP amino acid composition data compared well (within ±0.1–1.1% range) with the corresponding literature values [41]. These values also compared well (within ±0.02–0.76% range) with the amino acid composition reported for instantized nonfat dry milk powder [42].

The presented distribution of different amino acids in the authentic NFDM/SMP samples along with their corresponding lower and upper tolerance limits can be used as a reference amino acid fingerprint for authentic NFDM/SMP samples. These results are based on a large number of types of NDFMs/SMPs and take into account most the variation expected.

#### 3.2.1. Variance in Distribution of Different Amino Acids in Authentic NFDM/SMP Samples

The highest % RSD value of replicate NFDM/SMP analyses among amino acids was 11.5%, with the exception of that of His, which exhibited somewhat higher variation at 18.3% (Table 7). The latter could partially be a result of the lower distribution of His (2.7%) which happens to be in the lower range of all amino acids in NFDM/SMP. The fitting % RSD values for most the amino acids obtained for a large number and types of NFDM/SMP in the collaborative results can be indicative of close similarity in distribution of most of the amino acids in different samples. These values represent the variations due to differences in geographic origin and manufacturing processes, together with those associated with the method of the analysis.

#### 3.2.2. Assessment of Precision of the Amino Acid Analysis Method

The study attempted to evaluate the precision of the amino acid analysis method (% RSD). This was performed by analyzing amino acids in one of the NFDM samples (S091) in a higher number of replicates, i.e., 17–18 replicates by each laboratory with a total of 86 independent replicates. Analysis of amino acids using a large number of replicates of the same sample provides a correct estimate of precision of the analytical method. The average values of amino acid distribution in S091 and all of the NFDM/SMP samples, together with the corresponding % RSDs from analysis of S091 and all of the NFDM/SMP samples are compared in Table 8. These precision values are devoid of variations caused by other factors, i.e., different geographic origins and manufacturing processes, which also contribute to spread in values of each amino acid in all NFDM/SMP samples.

The averages of the distribution of amino acids in the sample S091 compared well with that of the corresponding overall mean of all NFDM/SMP samples and results were within ±0.08% for most of the amino acids. The average value of aspartic acid of S091 was lower by 0.13% as compared to the overall average of all the samples. The variation (% RSD) in replicate analyses of different amino acids in the S091 sample and all NFDM/SMP samples was similar for most of the amino acids and was within ±0.72% of the respective values for all NFDM/SMP samples. The % RSD for His in S091 was, however, considerably lower (4.46%) as compared to the respective value for all samples.

#### 3.2.3. Assessment of Variations in Amino Acid Distribution in NFDM/SMP Samples Caused by Differences in Their Geographic Origin and Manufacturing Processes

The results in Table 8 are helpful to understand the contribution of analytical variation in the overall variation observed in the distribution of amino acids in replicates of all authentic NFDM/SMP samples (Table 7). The analytical % RSD for His is considerably lower than that of variance in His distribution in different authentic samples, and in this regard, it follows a different pattern than other amino acids whose % RSD is close to that of analytical variance. This estimate indicates that His distribution in different NFDM/SMP samples is more sensitive to differences caused by geographic origin and manufacturing processes than other amino acids; these factors add to the analytical variance. The analytical variations observed for other amino acids in this evaluation are quite similar (±0.72%) to the variation in their distribution in the different NFDM/SMP samples.

### 3.3. Comparison of Amino Acid Composition of the Authentic NFDM/SMP Samples and Potential Adulterant Plant and Animal Proteins

**Figure 1:**
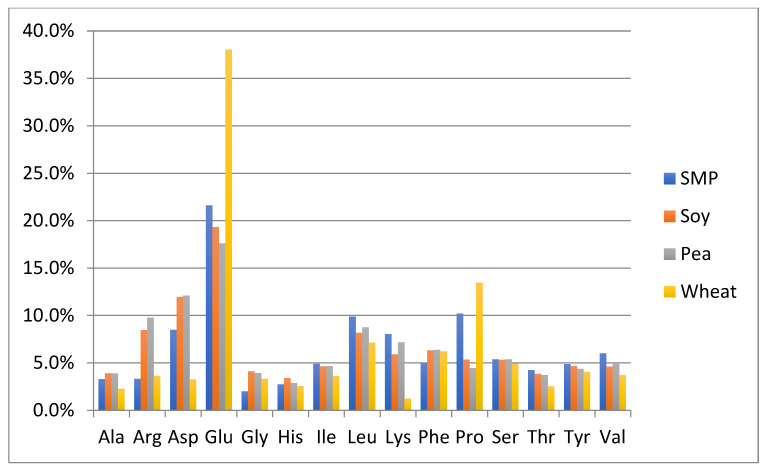
Comparison of amino acid distribution in NFDM/SMP samples with that of protein isolates of soy, pea, and wheat. Y axis = % of each amino acid (of the sum of all listed amino acids). SMP = skim milk powder, Soy = soy protein isolate, Pea = Pea protein isolate, Wheat = Wheat protein isolate.

**Figure 2:**
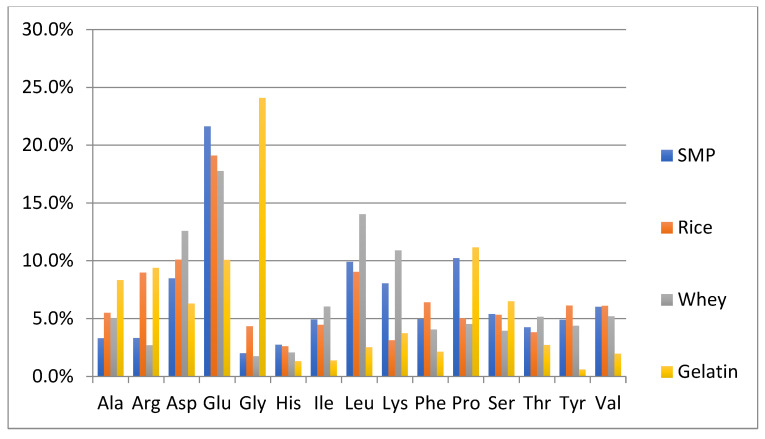
Comparison of amino acid distribution in NFDM/SMP samples with that of rice protein, whey and gelatin. Y axis = % of each amino acid (of the sum of all listed amino acids). SMP = skim milk powder, Rice = rice protein isolate, Whey = whey sample, Gelatin = Fish gelatin.

One of the objectives of this study was to perform a preliminary evaluation to detect differences in the amino acid composition between the authentic NFDM/SMP samples and some of the cheaper plant and animal proteins, which can potentially be used as adulterants. The adulterant proteins studied included slightly hydrolyzed soy protein isolate, pea protein isolate, hydrolyzed wheat protein isolate, rice protein isolate, whey protein isolate and high molecular weight fish gelatin. The soy and pea protein isolates were analyzed in duplicate; all other adulterant proteins were analyzed in triplicates. Amino acid analysis of these samples was performed by the same method as described for milk powders and those spiked with adulterants. The content of each amino acid is calculated as % of the sum of the contents of all analyzed amino acids in every potential adulterant as well as in NFDM/SMP samples. Average results of amino acid composition are presented in Figure 1 and Figure 2. Figure 1 provides a comparison of average values of amino acid distribution in milk powder samples with that of protein isolates of soy, pea, and wheat. Figure 2 provides a comparison of average values of amino acid distribution in NFDM/SMP samples with that of rice and whey protein isolates and fish gelatin.

Amino acids found to have a different distribution profile in different adulterants included at least one of the following: Ala, Arg, Asp, Glu, Gly, Lys and Pro. It may be added that Hyp was only detected in fish gelatin; it was not detected in any of the other potential adulterant proteins, nor was it detected in any of the NFDM/SMP and NIST samples or the milk powder samples spiked with the adulterants except fish gelatin at higher levels (0.3% and 0.6%). The presence of Hyp can be used as a marker for potential adulteration with fish gelatin.

Gly distribution was higher in all plant proteins and fish gelatin compared to milk powder. Arg was high in gelatin and plant proteins, except wheat. Ala was higher in rice, whey, and gelatin than in milk. Pro had a lower distribution in pea, rice soy, and whey compared to milk. Asp distribution was lower and Glu was higher in wheat protein. Lys was lower in wheat, rice, and gelatin. Gelatin was lower in additional amino acids including Tyr, Leu, Ile, Val, Phe and His.

The major differences in the amino acid distribution in the evaluated potential adulterant proteins as compared to NFDM/SMP are summarized in Table 9 and are consistent with literature reports describing the amino acid composition of these proteins [43]. The distribution of only those amino acids which are different by more than 50% of the corresponding values in the NFDM/SMP samples in at least one of the adulterants are listed in the following Table 9. The amino acids which were found to have different distribution in this regard in two or more potential adulterants included Gly, Arg, Ala, Lys, Pro and Glu.

The observed similarity in amino acid distribution in large number of authentic NFDM/SMP samples produced at different geographical locations by different manufacturing methods is important and encouraging and should be leveraged to authenticate NFDM and SMP samples. The resulting amino acid fingerprint thus can be used as one of the parameters to establish authenticity of NFDM/SMP samples. Considerable differences in the distribution of amino acids in the potential adulterants as compared to NFDM/SMP help further to illustrate the utility of using an amino acid fingerprint in authenticating milk powder samples and detecting adulteration. Studies were undertaken to determine the effects on the amino acid distribution of spiking NFDM/SMP samples with adulterants at different levels.

### 3.4. Effects of Spiking of NFDM/SMP Samples with Potential Adulterant on Amino Acid Composition

The amino acid composition of the authentic NFDM/SMP samples and those spiked with the potential adulterants was analyzed. The adulterants included plant and animal proteins as well as arginine and melamine and are listed along with their spiking levels in Table 2 in the Methods section. One of the NFDM/SMP samples (S091) was spiked with the listed adulterants at specified levels. The amino acid results of the spiked samples are compared to the corresponding values of non-spiked samples and are presented in Table 10 and Table 11. The distribution value of each amino acid is equal to the content of each amino acid calculated as % of the sum of the contents of all amino acids in the respective sample. The amino acid distribution data for the adulterant-spiked samples as the % of the corresponding values in non-spiked NFDM/SMP sample are presented in Table 12 and Table 13. 

The NFDM/SMP amino acid fingerprint was found to be affected by spiking with some adulterants at certain spiking levels. The significance of the differences of amino acid composition of the spiked samples from that of the non-spiked sample was tested by ANOVA and post hoc Dunnett’s test at *p* > 0.05.

Spiking of NFDM/SMP sample with melamine and wheat protein did not affect the distribution of any of its amino acids significantly at any of the spike levels. Melamine spiking is not expected to affect the amino acid profile of milk. Its spiking may cause a discrepancy between the measurement of total proteins in milk based on nitrogen content and that estimated by summation of all amino acids, but this evaluation was not performed in the current study. Distributions of some of the amino acids in the NFDM/SMP sample were affected by spiking with adulterants other than melamine and wheat protein, mostly at the highest spike levels.

The distribution of Gly in NFDM/SMP was increased significantly by spiking with pea or rice protein isolates at 2% levels (highest) and gelatin at 0.6% and 0.3% levels (highest and 2nd highest levels). Spiking with soy protein isolate also increased Gly distribution significantly at the 1% level while observed increases at other levels (including the 2% level) were of lesser magnitude and were not significantly different, probably due to analytical variability.

Spiking of NFDM/SMP with pea protein at 2% caused a significant increase in distribution of Phe and Tyr and a decrease of Lys.

Spiking with whey protein isolate at the highest spike level (3%) significantly increased distribution of Leu and decreased that of Pro. The distribution of Leu along with Thr increased at the 1.5% spike level. The increase in Thr distribution was not significant at other spike levels, including the 3% level.

Spiking of NFDM/SMP with Arg was also evaluated in the study because of its potential to be used for EMA due to its high nitrogen content. As expected, the distribution of Arg in milk protein amino acid fingerprint increased at the two highest spike levels evaluated (0.1% and 0.5%).

In general, the pattern of results of NFDM/SMP spiking with various adulterants is consistent with the major differences observed in amino acid composition of adulterants with that of milk powder (Figure 1 and Figure 2), i.e., higher levels of Gly in plant proteins and gelatin and higher levels of Leu in whey caused increases in the corresponding amino acids in NFDM/SMP after spiking. Some of the differences are not observed after spiking because of smaller levels of spiking employed or analytical variability and/or complex changes in distribution of amino acids in spiked samples.

#### Summary of Effects of Adulterant Spiking on Amino Acid Composition of NFDM/SMP Samples

The amino acid fingerprint of NFDM/SMP was affected by spiking with some adulterants at certain spiking levels, i.e., often at the highest and/or 2nd highest spiking level used. The distributions of amino acids significantly affected by spiking with adulterants are summarized in Table 14. Distributions of some of the amino acids like Gly were more sensitive to the spiking and were found to be affected by spiking with a wider variety of plant proteins such as pea or rice at the 2% spike level and soy at the 1% level. Spiking with fish gelatin also increased Gly at the two highest levels (0.6 and 0.3%). Spiking with whey protein affected the distribution of Pro and Leu at the 3% level (highest level); and Leu and Thr at 1.5% (2nd highest spiking level). Spiking with Arg caused an increase in its distribution at the two highest spike levels (0.1% and 0.5%).

The results of the spiking study demonstrate the utility of amino acid fingerprinting in detection of adulteration of NFDM/SMP samples. Some amino acids can be more helpful in detection of adulteration depending on the adulteration agents; spiking with plant proteins and gelatin were observed to cause an increase in Gly distribution. Spiking with whey similarly affected Leu, Pro and Thr. Additional studies may be helpful to evaluate the effects of more potential adulterants as well as adulteration using wheat protein at higher spike levels.

The results of spiking NFDM/SMP with various adulterants in the current study are based on the spiking of a single NFDM/SMP sample, as mentioned earlier. Similar adulterant spiking studies with various NFDM/SMP samples will be helpful not only to confirm the current findings, but also to know whether the sensitivity of these affects is changed by differences in the geographic origin and manufacturing processes associated with the milk. The results of the current study are important given the close similarity in amino acid composition of different NFDM/SMP samples, regardless of the geographic origin and manufacturing processes.

Amino acid fingerprinting analysis has been used previously to differentiate milk and non-milk proteins [25]. Our findings support the earlier reports of the utility of amino acid fingerprinting in authenticating milk proteins and detecting EMA when adulterants are present at high enough concentrations.

### 3.5. Amino Acid Ratio Calculations in Spiked and Non-Spiked Samples

Amino acid values calculated as % of sum of contents of all amino acids of spiked and non-spiked samples were further computed to understand the integrated effects of adulterant spiking on amino acids. The amino acids results of the NFDM/SMP and samples spiked with various adulterants were calculated as amino acid ratios (AAR) of the sum of the amino acids which showed a tendency to increase by adulterant spiking against valine values (which often decreased with adulterant spiking). The formula used to calculate amino acid ratios is as follows.

Amino Acid Ratio (AAR) value = 100 × ((Sum of 2 × Arg + Gly + 2 × His +2 × Tyr + 3 × Leu + Ser + Thr)/(4 × Val))

The multiplication constants in the calculations are used to improve the sensitivity of this metric in differentiating values of adulterant spiked samples from the non-spiked results. These were derived based preliminary evaluations of different constants.

The results are presented in Table 15 for non-spiked and adulterant spiked samples, but only those which caused a significant effect on AAR at least at one of their spike levels tested.

The amino acid ratio value of non-spiked samples was found to generally increase with adulterant spiking (except 0.05% Arg spike) (Table 15). Spiking with plant proteins increased the ratio in the range of 2.7 to 18.5, and spiking with gelatin and whey protein isolate increased the ratio in the range of 4.8 to 18.9. Arginine spiking (except 0.05% ) increased the AAR in the range of 1.9 to 16.6, and melamine spiking increased it in the smaller range of 1.1 to 4.5. The increase in AAR values observed with adulterant spiking was significantly different from that of the corresponding non-spiked values only in case of certain adulterants, i.e., pea protein isolate (2%), gelatin (0.3 & 0.6%), and arginine (0.5%). These adulterants affected the AAR only at the highest spike level tested, except in the case of fish gelatin, where the difference was also significant at the second highest spike level.

### 3.6. Multivariate Analysis

PCA and PLS-DA were performed on the whole data set. However, limited classification of possible adulterations was found. The PCA scores plot is shown in Figure S1, with each class surrounded with the respective confidence ellipse. The PLS-DA model yielded 55% correct adulteration by a 5-fold cross validation, with 5 latent variables used. It is worth noting that different pre-processing method and variable selections were tried for modelling; however, the results only have marginal fluctuations. Therefore, only the most typical result, i.e., the concentrations without any pre-processing, is presented here (Figures S1 and S2). The limited discrimination was possibly due to the fact that the levels of adulteration were relatively low in this study, and that the number of each adulterant sample was only around 1/10 of that of the authentic samples. The signals were submerged in huge amounts of uncollated multivariate signals. To demonstrate, the PLS scores with only adulterated samples at spiking levels >1% were also plotted in Figure S2. A better separation is shown. The data suggest that the multivariate model may work on samples with higher level of adulterations. However, based on the inter-laboratory results, we still focus on the prediction limit and amino acid ratios in the current manuscript.

## 4. Conclusions

The current study provides an amino acid fingerprint of authentic NFDM and SMP differing in geographic origin and manufacturing processes. The variance in the values of the distribution of each amino acid was used to calculate the lower and upper prediction tolerance limits of amino acid distribution in the authentic samples. The amino acid fingerprint and the corresponding prediction tolerance limits resulting from the current study can be useful in evaluating the authenticity of NFDM and SMP samples.

Some potential adulterants analyzed in the current study, including vegetable and animal proteins, showed a different amino acid distribution in comparison to the milk powder. The amino acids which often differed in their distribution in potential adulterant proteins in comparison to milk powder included Gly, Arg, Ala, Pro, Lys and Glu.

The NFDM/SMP amino acid fingerprint was found to be affected by spiking with some adulterants. Gly distribution was affected significantly by spiking with vegetable proteins such as soy, pea, or rice proteins and fish gelatin, but not with wheat protein. Pea protein also affected the distribution of Arg, Phe, Tyr and Lys and AAR. Whey protein spiking, on the other hand, affected distribution of Leu, Pro and Thr. Arg spiking impacted its levels, as well as the AAR. The latter was also affected by gelatin spiking. Melamine spiking, as expected, did not change the amino acid fingerprint of milk samples. The adulterants whose spiking significantly affected the distribution of amino acids in NFDM/SMP samples are summarized in Table 14.

The integrated effects of adulterant spiking on amino acid distribution in NFDM/SMP could be demonstrated by calculating AAR in adulterant spiked samples. The AAR values showed a tendency to increase with spiking, but it was significantly different from the non-spiked value only in the case of certain adulterants, i.e., pea protein isolate, fish gelatin, and arginine, at least in the samples spiked at the highest level.

The study describes a rapid, accurate and precise method for the determination of the amino acid fingerprint in milk powder samples. The results of the current study demonstrate the utility of amino acid fingerprinting as one tool to establish authenticity of NFDM/SMP samples and detect adulteration, particularly EMA. The amino acid fingerprint of authentic NFDM/SMP samples based on the results of the current study can be very helpful in this objective.

## Figures and Tables

**Table 1:** NFDM and SMP samples analyzed for amino acid composition in the study.

Sr. #	Sample Code	Sample Type	Process Type *	Sample Origin	Sample Protein Contents (% *w*/*w*)
1	S021	NFDM	LH	USA	35.67
2	S022	SMP	-	USA	33.71
3	S023	NFDM	MH	USA	35.4
4	S024	NFDM	MH	USA	35.56
5	S030	NFDM	HH	USA	-
6	S031	NFDM	HH	USA	-
7	S032 ***	NFDM	LH	USA	-
8	S033	NFDM	LH	USA	-
9	S047	NFDM	LH	USA	35.5
10	S053	NFDM	LH	USA	-
11	S054	NFDM	LH	USA	-
12	S055	NFDM	LH	USA	35.69
13	S068	NFDM	LH	USA	-
14	S070	NFDM	LH	USA	-
15	S076	NFDM	HH	USA	-
16	S077 ***	SMP	LH	USA	33.4
17	S080	SMP	LH	USA	34.29
18	S081	NFDM	HH	USA	35.44
19	S082	NFDM	LH	USA	36.09
20	S084	SMP	MH	USA	34.06
21	S086	NFDM	HH	USA	35.64
22	S087	NFDM	LH	USA	35.84
23	S089	NFDM	MH	USA	35.73
24	S091 ***	NFDM	MH	USA	36.31
25	S093	NFDM	LH	USA	36.04
26	S095 ***	SMP	MH	New Zealand	32.7
27	S096	SMP	MH	USA	34.12
28	S097	SMP	LH	USA	34.3
29	S106 ***	SMP	MH	Ireland	37
30	S107	SMP	MH	Ireland	35.7
31	S108	NFDM	-	-	-
32	S110	NFDM	-	-	-
33	S116 ***	SMP	MH	Denmark	-
34	S136	SMP		India	-
35	S145	NFDM	LH	USA	-
36	S147 ***	NFDM	LH	USA	-
37	S149	NFDM	HH	USA	-
38	S176 ***	SMP, Agglomerated		Argentina	-
39	S177 ***	NIST SRM 1549a Whole milk powder **			25.64

* Process Type: LH = Low Heat Spray Drying, MH = Medium Heat Spray Drying, HH = High Heat Spray Drying; - values not available. **—obtained from NIST, Gaithersburg, MD, USA. *** Core sample set tested by all laboratories.

**Table 2:** The adulterants and their spiking levels evaluated to see their effect on amino acid composition of NFDM/SMP.

Adulterant Sample Code	Adulterant Name and Abbreviation	Adulterant Spiking Levels (% *w*/*w*)
A002	Melamine (M)	0.03, 0.06, 0.16
A174	Slightly hydrolyzed soy protein isolate (S)	0.25, 0.50, 1.0, 2.0
A011	Pea protein isolate (P)	0.25, 0.50, 1.0, 2.0
A006	L-arginine (A)	0.01, 0.05, 0.10, 0.50
A028	Hydrolyzed wheat protein isolate (Wt)	0.25, 0.50, 1.0, 2.0
A019	Rice protein isolate (R)	0.25, 0.50, 1.0, 2.0
A046	Whey protein isolate (Wy)	0.30, 0.60, 1.5, 3.0
A013	High MW fish gelatin (G)	0.15, 0.30, 0.60

**Table 3:** Example of Hydrolysis Program for CEM instruments.

Apparatus	Discover SP	Discover SP-D
Power	200 watts	300 watts
Power Mode	Dynamic	Dynamic
Hold Time	15 min	15 min
Temperature	160°	160°
Pressure *	250 psi	250 psi
Pre-Stirring (high)	15 s	15 s
Power Max	Off	N/A
Blanket with Nitrogen	No	No

* Maximum pressure the instrument can reach before shutting down for safety purposes. This setting does not control the pressure of the system during hydrolysis which typically is in the 50 to 80 psi range.

**Table 4:** Gradient for Chromatography system A.

Time (min)	%A	%B	%C	%D	Gradient Curve *
0	10.0	0.0	90.0	0.0	Initial
0.29	9.9	0.0	90.1	0.0	11
5.49	9.0	80.0	11.0	0.0	7
7.10	8.0	15.6	57.9	18.5	6
7.30	8.0	15.6	57.9	18.5	6
7.69	7.8	0.0	70.9	21.3	6
7.99	4.0	0.0	36.3	59.7	6
8.59	4.0	0.0	36.3	59.7	6
8.68	10.0	0.0	90.0	0.0	6
10.20	10.0	0.0	90.0	0.0	6

* Gradient Curve: Curve 6 is a linear change over the segment, Curve 6 is a linear change over the segment and Curve 7 is a more shallow concave curve, while curve 11 is a step change at the end of the segment.

**Table 5:** Gradient for Chromatography system B.

Time (min)	%A	%B	Curve *
0	99.9	0.1	
0.54	99.9	0.1	6
5.74	90.9	9.1	7
7.74	78.8	21.2	6
8.04	40.4	59.6	6
8.05	10.0	90.0	6
8.64	10.0	90.0	6
8.73	99.9	0.1	6
9.50	99.9	0.1	6

* Gradient Curve: Curve 6 is a simple, straight linear change over the segment, and Curve 7 is a more shallow concave curve.

**Table 6:** Results of amino acid analysis in a casein sample (following hydrolysis by the microwave and a reference method ^@^).

Amino Acids	Reference Method		Microwave Method	
	Mean (*n* = 19) g/100 g as Is	% RSD	Mean (*n* = 5) g/100 g as Is	% RSD
Ala	2.57	4.3	2.68	3.6
Arg	3.41	6.9	3.46	4.8
Asp	6.62	3.7	7.11 *	2.7
Glu	19.77	4.2	20.62	2.8
Gly	1.67	4.7	1.76	3.9
His	2.51	8.3	2.54	2.8
Ile	4.31	13.4	4.46	1.9
Met	2.63	6.9	2.46	2.6
Leu	8.28	4.6	8.57	2.1
Lys	7.24	5.8	7.62	4.7
Phe	4.53	5.4	4.79	1.7
Ser	5.18	4.7	4.95	3.4
Thr	3.75	3.6	3.68	2.8
Tyr	4.98	7.9	5.16	1.1
Val	5.64	11.4	5.90	3.1
Total	83.09	4.1	86.01	3.8

Value marked with asterisk is significantly different from the respective reference value (*p* < 0.05). ^@^ Reference method of protein hydrolysis: AOAC method 982.30 [36]. Amino acid analyses in these studies were performed by MSS2 method of FSIS/USDA (1993) [37].

**Table 7:** Amino acid distribution (% of sum of all analyzed and displayed amino acids) in authentic NFDM/SMP samples.

Amino Acids	Mean	Std. Dev	Range of Distribution		*n*	% RSD	*k*	*k* × SD	Predicted Tolerance in Authentic NFDM/SMP	
			Min	Max					Lower Limit	Upper Limit
Ala	3.30%	0.16%	2.81%	3.98%	274	5.0%	1.972	0.33%	2.97%	3.62%
Arg	3.31%	0.34%	2.54%	4.43%	274	10.2%	1.972	0.66%	2.65%	3.98%
Asp	8.48%	0.70%	6.26%	10.39%	274	8.2%	1.972	1.38%	7.10%	9.86%
Glu	21.62%	1.19%	17.73%	24.98%	274	5.5%	1.972	2.36%	19.27%	23.98%
Gly	2.01%	0.15%	1.64%	2.43%	274	7.7%	1.972	0.30%	1.70%	2.31%
His	2.74%	0.50%	1.76%	4.96%	274	18.3%	1.972	0.99%	1.75%	3.73%
Ile	4.91%	0.31%	4.23%	5.96%	274	6.3%	1.972	0.61%	4.30%	5.52%
Leu	9.89%	0.19%	9.20%	10.58%	274	2.0%	1.972	0.38%	9.51%	10.27%
Lys	8.05%	0.75%	5.33%	10.13%	274	9.3%	1.972	1.48%	6.57%	9.53%
Phe	4.94%	0.56%	3.55%	7.37%	274	11.3%	1.972	1.10%	3.84%	6.03%
Pro	10.22%	0.27%	8.56%	11.10%	274	2.6%	1.972	0.53%	9.69%	10.75%
Ser	5.39%	0.25%	4.35%	6.05%	274	4.6%	1.972	0.49%	4.90%	5.87%
Thr	4.24%	0.11%	3.84%	4.66%	274	2.7%	1.972	0.22%	4.02%	4.46%
Tyr	4.89%	0.56%	3.50%	7.28%	274	11.5%	1.972	1.11%	3.78%	6.00%
Val	6.02%	0.41%	5.22%	7.04%	274	6.8%	1.972	0.81%	5.20%	6.83%

**Table 8:** Analytical variation vs. overall variation in amino acid composition of diverse NFDM/SMP samples.

Amino Acid	Average of S091 *	% RSD of S091 *	Average of All NFDM/SMP **	% RSD of All NFDM/SMP **	Difference in Mean of S091 and All NFDM/SMP	Difference in % RSD of S091 and All NFDM/SMP
Ala	3.25%	5.3%	3.30%	5.0%	−0.04%	0.31%
Arg	3.36%	9.8%	3.31%	10.2%	0.05%	−0.32%
Asp	8.35%	7.8%	8.48%	8.2%	−0.13%	−0.44%
Glu	21.57%	5.1%	21.62%	5.5%	−0.05%	−0.39%
Gly	2.01%	8.3%	2.01%	7.7%	0.00%	0.63%
His	2.76%	13.8%	2.74%	18.3%	0.02%	−4.46%
Ile	4.96%	5.9%	4.91%	6.3%	0.06%	−0.42%
Leu	9.89%	1.4%	9.89%	2.0%	−0.01%	−0.53%
Lys	8.05%	8.6%	8.05%	9.3%	−0.01%	−0.72%
Phe	5.01%	11.5%	4.94%	11.3%	0.08%	0.25%
Pro	10.19%	2.5%	10.22%	2.6%	−0.02%	−0.09%
Ser	5.34%	4.8%	5.39%	4.6%	−0.05%	0.19%
Thr	4.20%	2.5%	4.24%	2.7%	−0.03%	−0.13%
Tyr	4.96%	11.7%	4.89%	11.5%	0.07%	0.22%
Val	6.08%	6.4%	6.02%	6.8%	0.07%	−0.41%

* Each value is average of 86 analysis (*n* = 86); ** Each value is average of 274 analysis (*n* = 274); **%** RSDs of one of the NFDM/SMP samples analyzed by each lab in 17–18 replicates compared with analysis of all NFDM/SMP samples ** in the last column.

**Table 9:** Amino acid distribution in potential adulterant proteins in comparison to NFDM/SMP.

Commodity	Amino Acid Distribution as % of that in NFDM/SMP (Different More than 50%)						
	Ala	Arg	Asp	Glu	Gly	Lys	Pro
Soy	118	258 *	142	89	205 *	73	53
Pea	118	297 *	142	81	195 *	89	44 *
Wheat	70	109	39 *	176 *	165 *	15 *	132
Rice	167 *	273 *	119	88	215 *	38 *	49 *
Whey	152 *	82	148	82	85	135	44 *
Gelatin **	252 *	285 *	74	47 *	1205 *	46	110

Values marked with a single asterisk * are >50% different from corresponding NFDM.SMP values. ** Additional amino acids in gelatin have distributions differing more than 50% than for SMP/NFDM and those include, Ile, Leu, Lys, Phe, Tyr and Val.

**Table 10:** Amino acid composition of the NFDM/SMP samples with and without spiking with plant protein based on economically motivated adulterants at different spiking levels.

Samples AA-->	Ala	Arg	Asp	Glu	Gly	His	Ile	Leu	Lys	Phe	Pro	Ser	Thr	Tyr	Val
Unit-->	**Average of normalized values as % of sum of all listed amino acids (non-spiked, *n* = 86)**														
**Non-Spiked**	3.25	3.36	8.35	21.57	2.01	2.76	4.96	9.89	8.05	5.01	10.19	5.34	4.20	4.96	6.08
Spike %	**Pea protein isolate, A011 (P) *n* = 7 at each spike level)**														
0.25	3.31	3.35	8.34	21.37	1.98	2.80	4.97	9.96	8.01	4.99	10.22	5.39	4.26	5.00	6.06
0.50	3.29	3.40	8.26	21.22	2.03	3.08	4.98	9.93	7.90	5.07	10.17	5.36	4.22	5.07	6.02
1.00	3.29	3.47	8.33	21.24	2.06	2.81	5.00	9.96	7.93	5.06	10.15	5.40	4.26	5.01	6.05
2.00	3.17	3.81 *	7.74	19.92	2.37 *	3.16	5.05	10.09	7.07 *	5.73 *	10.24	5.54	4.30	5.73 *	6.08
Spike %	**Rice protein isolate, A019 (R) (*n* = 7 at each spike level except 0.5% spike *n* = 6)**														
0.25	3.30	3.46	8.19	21.17	2.07	2.72	5.00	9.98	7.79	5.12	10.32	5.42	4.27	5.16	6.03
0.50	3.32	3.38	8.35	21.33	2.07	2.83	4.82	9.90	7.76	5.16	10.28	5.53	4.33	5.13	5.82
1.00	3.32	3.47	8.31	21.38	2.02	2.81	5.00	9.91	7.88	5.07	10.10	5.37	4.27	5.05	6.04
2.00	3.31	3.62	8.10	20.81	2.17 *	3.02	4.97	9.92	7.45	5.39	10.13	5.43	4.27	5.40	6.01
Spike %	**Slightly hydrolyzed soy protein isolate, A174 (S) (*n* = 7 at each spike)**														
0.25	3.25	3.48	7.95	20.75	2.07	2.90	5.04	10.11	7.70	5.21	10.40	5.49	4.30	5.21	6.14
0.50	3.27	3.45	8.25	21.18	2.09	2.90	4.96	9.94	7.76	5.17	10.19	5.40	4.25	5.14	6.05
1.00	3.26	3.53	8.07	20.77	2.35 *	2.99	5.04	9.95	7.63	5.28	10.13	5.39	4.26	5.26	6.11
2.00	3.33	3.58	8.42	21.26	2.09	2.77	4.94	9.90	7.86	5.09	10.03	5.41	4.26	5.04	6.02
Spike %	**Hydrolyzed wheat protein isolate, A028 (Wt) (*n* = 6 at 0.25% spike level; *n* = 8 at 0.5% spike; *n* = 7 at 1% spike and 2% spike)**														
0.25	3.30	3.36	8.27	21.41	2.00	2.79	4.97	9.97	7.99	4.97	10.34	5.46	4.29	4.96	5.92
0.50	3.19	3.53	7.97	20.98	2.07	3.06	5.05	9.91	7.40	5.45	10.22	5.35	4.25	5.46	6.11
1.00	3.25	3.38	8.17	21.76	2.04	2.82	4.94	9.88	7.77	5.08	10.29	5.37	4.23	5.05	5.96
2.00	3.21	3.43	7.92	21.81	2.08	2.86	4.97	9.85	7.52	5.24	10.38	5.39	4.24	5.15	5.96

* Values marked with asterisk are significantly different from corresponding NFDM/SMP values at *p* < 0.05 based on ANOVA and post Dunnett’s test.

**Table 11:** Amino acid composition of the NFDM/SMP samples with and without spiking with animal proteins, arginine and melamine adulterants at different spiking levels.

Samples AA-->	Ala	Arg	Asp	Glu	Gly	His	Ile	Leu	Lys	Phe	Pro	Ser	Thr	Tyr	Val
Unit -->	**Average of normalized values as % of sum of all listed Amino acids (non-spiked, *n* = 86)**														
**Non-Spiked**	3.25	3.36	8.35	21.57	2.01	2.76	4.96	9.89	8.05	5.01	10.19	5.34	4.20	4.96	6.08
Spike %	**High MW fish gelatin, A013 (G) (*n* = 7 at 0.15 % spike level; *n* = 10 at 0.3 % spike; *n* = 12 at 0.6% spike)**														
0.15	3.29	3.37	8.23	21.20	2.12	2.80	4.99	9.89	7.96	5.06	10.19	5.33	4.25	5.06	6.02
0.30	3.34	3.41	8.26	21.21	2.22 *	2.87	4.85	9.87	7.84	5.12	10.32	5.46	4.28	5.11	5.84
0.60	3.37	3.52	8.10	20.94	2.45 *	2.85	4.92	9.87	7.78	5.07	10.36	5.49	4.30	5.04	5.92
Spike %	**Whey protein isolate, A046 (Wy) (*n* = 7 at each spike level except 0.3% spike level with *n* = 6)**														
0.30	3.33	3.32	8.41	21.37	2.00	2.76	4.92	9.97	8.07	4.96	10.27	5.46	4.31	4.96	5.88
0.60	3.33	3.36	8.35	21.33	1.98	2.85	5.00	9.98	8.06	4.98	10.14	5.34	4.28	4.99	6.03
1.50	3.33	3.41	8.28	21.01	2.04	2.90	5.04	10.12 *	7.83	5.12	10.06	5.39	4.35 *	5.13	6.00
3.00	3.29	3.36	8.36	21.07	1.94	2.80	4.97	10.21 *	8.55	5.01	9.84 *	5.32	4.25	5.00	6.05
Spike %	**L-arginine, A006 (A) (*n* = 8 at 0.01 % spike level; *n* = 7 at 0.05% spike; *n* = 6 at 0.1% spike and *n* = 7 at 0.5% spike)**														
0.01	3.30	3.39	8.31	21.47	2.00	2.75	5.01	9.94	8.06	4.95	10.23	5.34	4.23	4.97	6.06
0.05	3.27	3.56	8.15	21.21	2.00	2.78	5.09	9.96	8.00	5.02	10.21	5.35	4.27	5.02	6.11
0.10	3.29	3.66 *	8.23	21.40	1.95	2.71	5.01	9.93	8.04	4.97	10.18	5.36	4.25	4.97	6.05
0.50	3.24	4.71 *	8.18	21.10	2.05	2.76	4.92	9.78	7.90	4.94	10.06	5.28	4.19	4.96	5.94
Spike %	**Melamine, A002 (M) (*n* = 7 at each spike)**														
0.03	3.18	3.41	8.18	21.66	1.91	2.84	4.91	10.00	8.13	4.97	10.03	5.41	4.18	5.07	6.11
0.06	3.28	3.34	8.23	21.30	2.03	2.78	4.97	9.96	7.92	5.12	10.22	5.36	4.22	5.20	6.05
0.16	3.30	3.32	8.34	21.43	1.98	2.81	4.97	9.93	8.06	4.96	10.24	5.35	4.25	4.98	6.08

* Values marked with asterisks are significantly different from corresponding NFDM/SMP values at *p* < 0.05 based on ANOVA and post Dunnett’s test.

**Table 12:** Amino acid composition results of NFDM/SMP samples spiked with plant protein based on economically motivated adulterants at different spiking levels presented as % of corresponding non-spiked values.

Samples AA-->	Ala	Arg	Asp	Glu	Gly	His	Ile	Leu	Lys	Phe	Pro	Ser	Thr	Tyr	Val
Unit-->	**Average of normalized results as % of respective non-spiked value**														
Non-Spiked	100.0	100.0	100.0	100.0	100.0	100.0	100.0	100.0	100.0	100.0	100.0	100.0	100.0	100.0	100.0
Spike %	**Pea protein isolate, A011 (P) *n* = 7 at each spike level)**														
0.25	101.7	99.6	99.8	99.1	98.2	101.5	100.0	100.7	99.6	99.6	100.2	101.1	101.4	100.8	99.6
0.50	101.1	100.9	98.9	98.4	100.9	111.5	100.4	100.4	98.2	101.2	99.8	100.4	100.5	102.3	99.0
1.00	101.2	103.0	99.7	98.5	102.6	101.7	100.6	100.8	98.5	100.9	99.6	101.2	101.2	100.9	99.5
2.00	97.5	113.1 *	92.7	92.4	118.0 *	114.6	101.6	102.1	87.8 *	114.3 *	100.5	103.8	102.3	115.4 *	100.0
Spike %	**Rice protein isolate, A019 (R) (*n* = 7 at each spike level except 0.5% spike level with *n* = 6)**														
0.25	101.4	102.9	98.0	98.1	103.1	98.7	100.6	101.0	96.9	102.1	101.2	101.5	101.5	103.9	99.2
0.50	102.0	100.3	100.0	98.9	103.0	102.6	97.1	100.1	96.5	102.9	100.8	103.7	102.9	103.5	95.6
1.00	102.0	103.2	99.4	99.1	100.2	101.9	100.7	100.3	97.9	101.1	99.1	100.6	101.6	101.8	99.3
2.00	101.7	107.5	97.0	96.5	108.1 *	109.3	100.1	100.4	92.5	107.6	99.4	101.8	101.5	108.8	98.7
Spike %	**Slightly hydrolyzed soy protein isolate, A174 (S) (*n* = 7 at each spike level)**														
0.25	99.9	103.5	95.2	96.2	102.7	105.0	101.5	102.3	95.7	104.0	102.0	102.9	102.3	104.9	100.9
0.50	100.6	102.4	98.8	98.2	103.7	105.0	100.0	100.5	96.5	103.1	99.9	101.3	101.1	103.7	99.4
1.00	100.2	104.9	96.6	96.3	116.8 *	108.3	101.5	100.6	94.8	105.4	99.4	101.0	101.3	105.9	100.4
2.00	102.4	106.3	100.8	98.6	104.0	100.5	99.6	100.2	97.7	101.6	98.3	101.3	101.2	101.5	98.9
Spike %	**Hydrolyzed wheat protein isolate, A028 (Wt) (*n* = 6 at 0.25% spike level; *n* = 8 at 0.5% spike; *n* = 7 at 1% spike and 2% spike levels)**														
0.25	101.5	99.8	99.0	99.3	99.6	101.0	100.1	100.8	99.4	99.2	101.4	102.3	102.0	99.9	97.3
0.50	98.2	104.9	95.4	97.3	102.8	110.7	101.7	100.3	91.9	108.7	100.2	100.3	101.0	110.0	100.5
1.00	100.0	100.5	97.8	100.9	101.2	102.2	99.5	99.9	96.6	101.4	101.0	100.6	100.7	101.7	98.0
2.00	98.7	102.0	94.8	101.1	103.2	103.6	100.0	99.7	93.4	104.5	101.8	101.0	100.8	103.7	98.0

* Values marked with asterisk are significantly different from corresponding NFDM/SMP value at *p* < 0.05 based on ANOVA and post Dunnett’s test.

**Table 13:** Amino acid composition results of NFDM/SMP samples spiking with animal protein, arginine and melamine adulterants at different spiking levels presented as % of corresponding non-spiked values.

Samples AA-->	Ala	Arg	Asp	Glu	Gly	His	Ile	Leu	Lys	Phe	Pro	Ser	Thr	Tyr	Val
Unit-->	**Average of normalized results as % of respective non-spiked values**														
**Non-Spiked**	100.0	100.0	100.0	100.0	100.0	100.0	100.0	100.0	100.0	100.0	100.0	100.0	100.0	100.0	100.0
Spike %	**High MW fish gelatin, A013 (G) (*n* = 7 at 0.15% spike level; *n* = 10 at 0.3 % spike; *n* = 12 at 0.6% spike)**														
0.15	101.3	101.8	98.5	98.3	105.8	101.5	100.5	100.0	99.0	100.9	100.0	100.0	101.1	101.9	99.0
0.30	102.5	101.2	98.9	98.3	110.5 *	104.1	97.6	99.8	97.4	102.2	101.2	102.3	101.8	103.0	96.0
0.60	103.6	104.5	97.0	97.1	121.5 *	103.2	99.0	99.9	96.7	101.2	101.6	102.9	102.3	101.6	97.3
Spike %	**Whey protein isolate, A046 (Wy) (*n* = 7 at each spike level except 0.3% spike level with *n* = 6)**														
0.30	102.2	98.8	100.7	99.1	99.6	99.9	99.0	100.9	100.3	99.0	100.7	102.3	102.6	100.0	96.7
0.60	102.3	99.9	100.0	98.9	98.5	103.3	100.7	101.0	100.1	99.4	99.5	100.1	101.8	100.5	99.1
1.50	102.4	101.3	99.2	97.4	101.3	104.9	101.5	102.3 *	97.3	102.1	98.7	101.0	103.5 *	103.3	98.6
3.00	101.0	99.9	100.1	97.7	96.2	101.3	100.1	103.3 *	106.3	100.0	96.5 *	99.6	101.1	100.7	99.5
Spike %	**L-arginine, A006 (A) (*n* = 8 at 0.01 % spike level; *n* = 7 at 0.05% spike; *n* = 6 at 0.1% spike and *n* = 7 at 0.5% spike)**														
0.01	101.5	100.8	99.5	99.5	99.2	99.6	100.9	100.5	100.2	98.7	100.3	100.2	100.6	100.1	99.6
0.05	100.6	105.7	97.6	98.3	99.4	100.8	102.4	100.8	99.4	100.2	100.1	100.2	101.5	101.2	100.5
0.10	101.2	108.8 *	98.5	99.2	96.7	98.1	101.0	100.5	99.9	99.2	99.9	100.5	101.0	100.1	99.5
0.50	99.6	140.1 *	97.9	97.8	101.8	100.0	99.1	98.9	98.2	98.6	98.7	99.0	99.8	99.9	97.6
Spike %	**Melamine, A002 (M) (*n* = 7 at each spike level)**														
0.03	97.7	101.3	98.0	100.4	95.0	103.0	98.8	101.1	101.0	99.2	98.4	101.5	99.5	102.3	100.4
0.06	101.0	99.4	98.5	98.8	100.9	100.8	100.1	100.7	98.4	102.1	100.3	100.5	100.3	104.9	99.5
0.16	101.5	98.8	99.9	99.4	98.6	101.6	100.1	100.5	100.2	98.9	100.5	100.4	101.0	100.3	99.9

* Values marked with asterisk are significantly different from corresponding non-spiked NFDM/SMP value at *p* < 0.05 based on ANOVA and post Dunnett’s test.

**Table 14:** Summary of Adulterant spiking effects on NFDM/SMP Amino Acid Composition.

Adulterant	Spiking Level	Amino Acids Significantly Affected
Pea Protein	2% (highest)	Gly, Arg, Phe, Tyr, Lys *, AAR
Rice Protein	2% (highest)	Gly
Soy Protein	1% (2nd highest)	Gly
Whey Protein	3% (highest)	Leu, Pro *
	1.5% (2nd highest)	Leu, Thr
Gelatin	0.6% (highest), 0.3% (2nd highest)	Gly, AAR
Arg	0.5% (highest)	Arg, AAR
	0.1% (2nd highest)	Arg

Listed amino acids are significantly different from corresponding non-spiked NFDM/SMP at value *p* < 0.05 based on ANOVA and post Dunnett’s method. Distribution of all amino acids as well as AAR (amino acid ratio) values were higher than that of NFDM/SMP except those marked with * providing lower values.

**Table 15:** Amino acid ratio (AAR) results of NFDM/SMP samples and those spiked with certain potential adulterants.

Samples	Ratios of Amino Acids
Non-Spiked	263.3 ± 13.7
Pea protein isolate spike 0.25%	263.9 ± 12.6
Pea protein isolate spike 0.50%	268.4 ± 17.4
Pea protein isolate spike 1.0%	265.8 ± 15.2
Pea protein isolate spike 2.0%	279.7 ± 24.4 *
Fish Gelatin spike 0.15%	265.3 ± 17.1
Fish Gelatin spike 0.30%	276.1 ± 14.0 *
Fish Gelatin spike 0.60%	273.9 ± 14.6 *
Arginine Spike 0.01%	263.0 ± 13.7
Arginine Spike 0.05%	262.2 ± 13.4
Arginine Spike 0.10%	265.2 ± 15.7
Arginine Spike 0.50%	277.4 ± 13.7 *

Amino acid ratios calculated based on values of individual amino acid as % of sum of all amino acids. Amino Acid Ratio value = 100 ((Sum of 2 × Arg + Gly + 2 × His +2 × Tyr + 3 × Leu + Ser + Thr)/4 × Val). * Values marked with asterisk are significantly different from corresponding NFDM/SMP at value *p* < 0.05 based on ANOVA and post Dunnett’s method.

## Data Availability

The data that support the findings of this study are available from the corresponding author at reasonable request.

## Supplementary Materials

The following supporting information can be downloaded at: https://www.mdpi.com/article/10.3390/foods11182868/s1, Figure S1: Principal component scores plot of authentic and adulterated samples. The data were directly used without further pretreatments. Each class was surrounded with its own confidence ellipses (*p* < 0.05). Figure S2: Principal component scores plot of authentic and selected adulterated samples with spiking levels > 1%. The data were directly used without further pretreatments. Each class was surrounded with its own confidence ellipses (*p* < 0.05).
