# Supplementary material for: Amino Acid Fingerprinting of Authentic Nonfat Dry Milk and Skim Milk Powder and Effects of Spiking with Selected Potential Adulterants
Source: Foods. 2022 Sep 16;11(18):2868. doi: 10.3390/foods11182868 (PMC9498471; doi:10.3390/foods11182868)
Supplement: Supplementary file 1 [file foods-11-02868-s001.zip › foods-1843184-supplementary.pdf]

Supplementary Materials

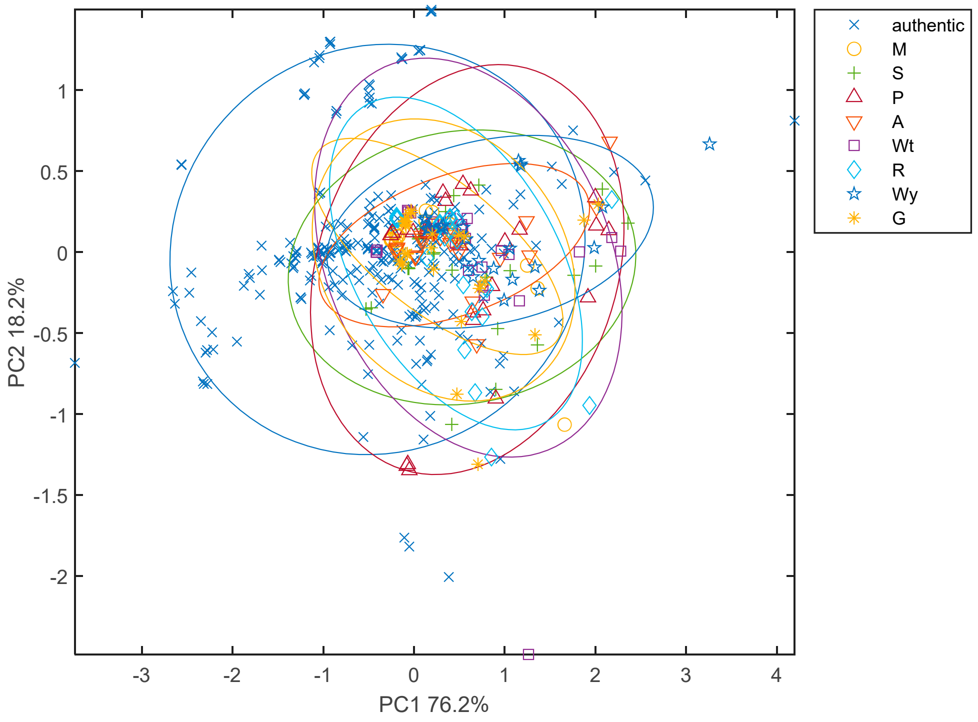

Figure S1. Principal component scores plot of authentic and adulterated samples. The data were directly used without further pretreatments. Each class was surrounded with its own confidence ellipses ($p < 0.05$).

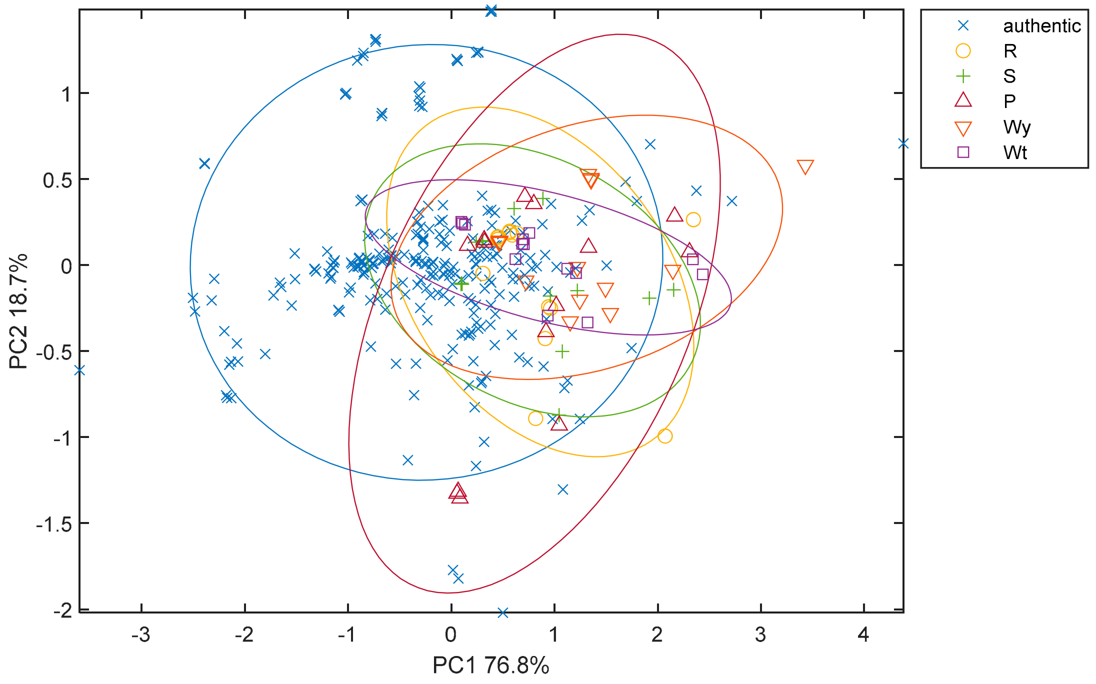

Figure S2. Principal component scores plot of authentic and selected adulterated samples with spiking levels > 1%. The data were directly used without further pretreatments. Each class was surrounded with its own confidence ellipses ($p < 0.05$).
